# Evaluation, Identification, and Management of Acute Methotrexate Toxicity in High-dose Methotrexate Administration in Hematologic Malignancies

**DOI:** 10.7759/cureus.2040

**Published:** 2018-01-08

**Authors:** Doron Feinsilber, Roberto J Leoni, Duminda Siripala, Julianne Leuck, Katrina A Mears

**Affiliations:** 1 Hematology/Oncology, Medical College of Wisconsin/Froedert Cancer Center; 2 Osteopathic Medicine, Nova Southeastern University School of Osteopathic Medicine; 3 Nephrology, UPMC Altoona; 4 Department of Ophthalmology, Retina Consultants of Southwest Florida, National Ophthalmic Research Institute, Fort Myers, Fl

**Keywords:** methotrexate, acute renal failure, neurotoxicity, glucoparidase, ocular oncology, ocular lymphoma, cns malignancies, primary cns lymphoma, lymphoma

## Abstract

The pharmacological and medical management of complex chemotherapy regimens are vast and complex, requiring an intimate understanding of physiology, particularly when novel biologic agents are utilized with commonly used regimens. The molecular classification in patients with diffuse large B-cell lymphoma (DLBCL) is multifaceted, particularly with the expansion of novel molecular targets. The pharmacological and medical management of hematologic malignancies with a tendency to have central nervous system (CNS) involvement is complex and requires an understanding of physiology and pharmacology. Many chemotherapy regimens used to treat hematologic malignancies with either CNS involvement or high risk for CNS disease will include the administration of high dose methotrexate. This requires having physiological understanding with respect to the standard regimens for DLBCL in addition to understanding cytogenetic markers, such as c-myc and bcl-2, the expression of which displays increased likelihood of CNS involvement. In patients with documented CNS disease and active neurological manifestations such as myclonus, headaches, nystagmus, and blurred vision, the utilization of high dose methotrexate has become an essential standard of care. We examine the pharmacologic mechanisms of high dose methotrexate in patients with hematologic malignancies such as DLBCL and review the most common toxicities on a multidisciplinary level.

## Introduction and background

Pharmacology and metabolism

In order to have a more complete concept of the multifactorial aspects of methotrexate toxicity, we must first characterize the pharmacologic mechanism of the action of methotrexate (MTX). MTX is an antifolate metabolite that inhibits dihydrofolate reductase (DHFR). DHFR subsequently reduces the conversion of folate to dihydrofolate (DHF) and DHF to tetrahydrofolate (THF)[1]. MTX enters the cell via the reduced folate carrier, where it undergoes polyglutamation and inhibits DHFR, depleting cells of reduced tetrahydrofolate cofactors. The accumulation of MTX polyglutamates and the increased concentration of dihydrofolates result in the blockage of de novo nucleotide synthesis. Early on we can already identify a potential therapeutic target in acute toxicity. These polyglutamates later will be examined in greater detail with respect to treatment of acute toxicity. Other enzymes such as thymidylate synthase, glycinamide ribonucleotide synthetase, and aminoimidazole-4 carboxamide ribonucleotide (AICAR) transformylase are involved in the latter components of the purine biosynthetic pathway.

With respect to MTX first-pass metabolism, approximately 80-90% of the drug is excreted unchanged in the urine. To a degree, some hepatic component plays a pivotal role in MTX clearance. Hepatotoxicity is well characterized by the National Cancer Institute (NCI) through the "Common Toxicity Criteria for Adverse Events" guidelines. These guidelines factor in elevations in hepatic enzymes including aspartate aminotransferase (AST), alanine aminotransferase (ALT), gamma-glutamyltransferase (GGT), and alkaline phosphatase (ALP) into: mild being 1–2.5 times the upper limit of normal (ULN), moderate being 2.5–5 times the ULN, severe being 5–20 times the ULN, and life-threatening being greater than 20 times the ULN. With hepatic insufficiency, we must evaluate factors such as acquired coagulopathies and hepatic clearance for other complex drugs regimens including cyclophosphamide and cytarabine.

## Review

Acute nephrotoxicity - evaluation, identification, and medical management

The etiology of MTX-induced renal dysfunction is mediated by the precipitation of MTX and its metabolites in the renal tubules, particularly with high-dose intravenous MTX, causing direct tubular injury [1-3]. The risk of MTX-induced nephrotoxicity is increased with acidic urine, as MTX is poorly soluble in low pH, leading to intratubular MTX crystallization and obstruction of urine flow. For this very reason, intravenous bicarbonate is traditionally built into therapy plans with a target pH of eight prior to administration of the drug. As will be discussed later, the risks for systemic side effects are significant in patients with intravascular volume depletion, which reduces urine flow rate and increases the concentration of MTX in tubular fluid. MTX that is poorly cleared can have an additive effect on compromising the glomerular filtration rate (GFR) by inducing afferent arteriolar constriction or mesangial cell constriction [4]. In a broader pharmacologic sense, a complete medication review as part of a root-mean analysis is necessary. In particular, we must determine if the patient had recent exposure to common drugs such as proton pump inhibitors (PPIs) and trimethoprim/sulfamethoxazole (TMP/SMX) as these commonly used medications can significantly decrease drug clearance synergistically. Careful attention must be taken in patients with a suspected pleural effusion as potential third-spacing of MTX could result in poor drug clearance and increase the additional risk for developing chemical pneumonitis.

The majority of patients with renal dysfunction are initially asymptomatic, and most present with nonoliguric renal dysfunction, indicated by an abrupt rise in serum creatinine during or shortly after MTX infusion [2]. Some early signs of neurotoxicity can include nystagmus and myoclonus, to name a few. In all cases the patient must have a complete neurological work up including evaluation of leptomeningeal spread by lumbar puncture and cerebrospinal fluid (CSF) studies in addition to electroencephalogram (EEG) and frequent bedside neurological assessments including seizure precautions. The administration of high doses of intravenous MTX permits high drug concentrations to be achieved within the central nervous system, which has been shown to result in acute, subacute, and long-term neurotoxicity. The precise mechanism of neurotoxicity is likely through disruption of CNS folate homeostasis and/or direct neuronal damage [5]. Aseptic meningitis symptoms may include symptoms such as headaches, nuchal rigidity, back pain, nausea, fatigue, vomiting, fever, and lethargy [6]. From a systems perspective, careful institutional attention must be taken with respect to pharmacy order sets that have specific protocol for infusion of folinic acid or otherwise known as leucovorin. The revised International Prognostic Index (R-IPI) was developed to predict the outcome of individuals receiving rituximab with standard chemotherapy such as high dose methotrexate. The score is able to differentiate patients into three groups (very good, good, poor), all of who have survival greater than fifty percent in the current era.

Identification, evaluation, and management of high-dose methotrexate

A common issue with administration of high-dose methotrexate (MTX) is nephrotoxicity, which may occur through both precipitation in the renal tubules and direct toxicity to the tubules [1]. For example, a case report of a 52-year-old male receiving intravenous high-dose methotrexate for aggressive DLBCL demonstrated the presence of methotrexate-like crystals in the urine. A renal biopsy performed seven days following the infusion demonstrated intratubular and interstitial deposition of numerous needle-shaped golden crystals arranged in annular structures [2]. This implies that methotrexate causes acute kidney injury through obstruction of the renal tubules. Also, a possible mechanism for direct toxicity to the renal tubules caused by methotrexate may involve oxidative stress. For example, a study in adult male Wistar rats determined that rats treated with methotrexate and caffeic acid phenylethyl ester, an antioxidant, showed reduced production of malondialdehyde (MDA), a breakdown product of polyunsaturated fatty acids in comparison with those rats treated solely with methotrexate. Also, the rats treated solely with methotrexate showed significantly higher production of MDA in comparison with control rats, who did not receive methotrexate. The same study also compared activity of superoxide dismutase, a scavenger of reactive oxygen species, among the control rats, the rats treated only with methotrexate, and the rats treated with both methotrexate and caffeic acid and phenylethyl ester. Superoxide dismutase activity was highest in the control group (64.38 U/g protein), lowest in the group receiving only methotrexate (42 U/g protein), and second-highest in the group treated with both methotrexate and caffeic acid and phenylethyl ester (50 U/g protein) [3]. The study not only suggests the potential role of lipid peroxidation in the renal tubules caused by methotrexate but also implies a potential preventative or curative role of antioxidant administration before or during administration of high-dose methotrexate.

Acute kidney injury resulting from administration of high-dose methotrexate generally presents with nonoliguric renal dysfunction characterized by an abrupt rise in serum creatinine levels during or shortly following methotrexate infusion, along with mucositis, hepatotoxicity, nausea, vomiting, and diarrhea [1]. Uremia from acute MTX toxicity can stimulate the chemoreceptor trigger zone (CTZ) and result in stimulation of the vagosympathetic nervous system and ultimate stimulation of the emetic center. Nausea and vomiting from uremic stimulation of the CTZ can result in acute intravascular volume depletion, further propagating renal toxicity of MTX. As such hydration status must be carefully monitored and titrated, particularly in an inpatient setting. Due to continued precipitation in the renal tubules, methotrexate cannot be effectively excreted. This is a significant issue because methotrexate is 90% renally cleared [1]. Therefore, patients developing acute kidney injury as a result of high-dose methotrexate administration are more likely to develop the typical adverse effects, such as myelosuppression, mucositis, hepatotoxicity, and chemical pneumonitis.

The most standard and current management of methotrexate-induced nephrotoxicity include intravenous (IV) fluid hydration, alkalinization of the urine, and leucovorin rescue. Alkalinization of the urine is employed because methotrexate and its metabolites, 7(OH) methotrexate and DAMPA, are poorly soluble at acidic pH and are much more soluble at a urine pH ranging from six to seven. Therefore, the current recommendation for IV fluid hydration calls for administration at a rate of 2.5–3.5 liters/m^2^ over two hours, beginning 12 hours prior to infusion of high-dose methotrexate and ending 24–48 hours afterward. In addition, current guidelines recommend addition of 40–50 mEq of sodium bicarbonate to each liter of intravenous fluid [1]. This allows dissolution and flushing of the methotrexate crystals present in the renal tubules. In addition, leucovorin rescue is recommended because leucovorin competes with methotrexate to enter cells through the reduced folate carrier. Once leucovorin enters the cells, it undergoes conversion to five-methyltetrahydrofolate, which allows replenishment of intracellular folate. Methotrexate inhibits dihydrofolate reductase, leading to a reduction in folate production [1]. Essentially, leucovorin antagonizes methotrexate through enhancement of further production of folate, which ameliorates potential adverse effects such as myelosuppression, hepatotoxicity, and GI side effects, which most likely occur as a result of inhibition of cell division caused by reduced intracellular folate levels.

In spite of these measures, however, renal dysfunction has been noted to occur in 1.8% of patients treated with high-dose methotrexate [1]. Therefore, other measures have been considered. Once such measure involves administration of glucoparidase, which is a carboxypeptidase G enzyme isolated from Pseudomonas. This enzyme hydrolyzes the terminal glutamate residue of methotrexate and its metabolites. This leads to the formation of 2,4 diamino-N10 methylpteroic acid (2,4 DAMPA) and OH-DAMPA, which are partially metabolized by the liver and  extrarenally eliminated [1,4]. A pooled analysis from four multicenter open-label clinical trials recently demonstrated the efficacy of glucoparidase. The trials enrolled patients who had developed acute kidney injury as a result of methotrexate-induced nephrotoxicity. The study revealed that 87% of the patients experienced a 95% or greater reduction in serum methotrexate concentrations a median of five minutes following glucoparidase administration. Also, 98% of the patients with serum methotrexate concentrations greater than or equal to 50 umol/L prior to glucoparidase administration and 83% of the patients with concentrations less than 50 umol/L experienced 95% reductions in serum methotrexate levels at the first measurements following treatment with glucoparidase. This also translated into recovery of renal function. Four hundred and thirty-six of the patients in the analysis experienced a three and a half fold rise in serum creatinine levels, on average from 0.79 to 2.79 mg/dl (reference range 0.3-1.0 mg/dl) following administration of high-dose methotrexate. On average following  glucoparidase administration, serum creatinine initially rose by 0.24 mg/dl, then began to decline by four days afterwards. Twenty-one days following glucoparidase administration, serum creatinine had decreased to 1.27 mg/dl in 148 of the original 436 patients. Also, 257 of the original 436 patients achieved a serum creatinine of 1.7 mg/dl following  glucoparidase administration [4]. 

While the study certainly demonstrates that glucoparidase leads to extrarenal metabolism of methotrexate and also suggests the potential benefits of glucoparidase administration with regard to recovery of renal function, another question may involve the timing of glucoparidase administration. Possible areas for further research may include early versus delayed administration of glucoparidase and the likelihood of further recovery of renal function. This is important as, in many cases, acute kidney injury becomes irreversible after a certain point although that point, as of yet, is unknown. For example, delayed administration of intravenous fluid hydration more often than not leads to ischemic acute tubular necrosis (ATN), in which case renal function is slower to recover and less likely to completely resolve. In fact, generally patients who have developed acute kidney injury are more prone to the development of chronic kidney disease. Another possible area for future investigation may include a cohort study comparing patients who received standard management of methotrexate nephrotoxicity, including IV fluid hydration, urinary alkalinization, and leucovorin rescue, and patients who received glucoparidase and the standard management. The study would compare the incidence of chronic kidney disease in patients who had previously received glucoparidase in addition to the standard management of methotrexate nephrotoxicity and those who only received standard management.

In the past, intermittent hemodialysis has been suggested as a possible management strategy for methotrexate-induced nephrotoxicity. However, it has been noted to be ineffective due to the fact that methotrexate has a large volume of distribution, which is mainly intracellular, allowing rebound in serum methotrexate levels following conventional hemodialysis [1,4]. Intermittent hemodialysis is more effective for removing molecules distributing primarily in plasma. An exception may include high-flux hemodialysis. Several cases in the literature have documented reduction in serum methotrexate levels and also recovery of renal function. For example, one case involved a 20-year-old male with pre T-cell acute lymphoblastic leukemia (ALL) who had developed a rise in serum creatinine levels to 8.3 mg/dL three days following administration of high-dose methotrexate. In addition, he had developed facial puffiness, pedal edema, and vomiting. As glucoparidase was unavailable, and the patient was demonstrating symptoms suggestive of volume overload and uremia, leucovorin rescue and high-flux hemodialysis were initiated. High-flux hemodialysis was performed for four hours on a daily basis. Ten days following initiation of high-flux hemodialysis, the serum creatinine had improved to 1.5 mg/dL [5].

Another case report discusses the use of high-flux hemodialysis in a 26-year-old male who had developed CNS lymphoma as a result of post-transplant lymphoproliferative disorder. The patient had received high-dose methotrexate (4 g/m^2^ ), leucovorin (30 mg/m^2^), and rituximab (500 mg/m^2^). His baseline glomerular filtration rate (GFR) was 20 mL/min/1.72 m^2^ and his serum methotrexate level 30 umol/L. After the patient had received four daily sessions of high-flux hemodialysis, his serum methotrexate levels had dropped to 0 umol/L and his GFR had not dropped significantly [6]. These case reports demonstrate not only plasma clearance of methotrexate using high-flux hemodialysis, but also recovery of renal function. However, as these studies are based on individual cases, it is difficult to say whether high-flux hemodialysis should be recommended as a core strategy for management of methotrexate-induced nephrotoxicity. However, it is likely that high-flux hemodialysis was more effective in removing methotrexate than conventional intermittent hemodialysis for several reasons. High-flux hemodialysis uses larger pores in the filters, which allows more effective clearance of larger molecules such as methotrexate in comparison with conventional intermittent hemodialysis. Furthermore, the high-flux hemodialysis was performed on a daily basis, unlike intermittent hemodialysis, which was performed every other day. This allowed less time for rebound in serum methotrexate levels leading to more efficient clearance of serum methotrexate. However, the idea that high-flux hemodialysis facilitated recovery of renal function is questionable. While methotrexate did originally cause acute kidney injury (AKI) and it is very likely that high-flux hemodialysis did provide effective clearance of the drug, hemodialysis itself can often further precipitate AKI by causing hypotension during treatment, leading to potential renal hypoperfusion. Therefore, more highly-powered studies examining initiation of high-flux hemodialysis and recovery of renal function need to be done, in order to determine whether in fact high-flux hemodialysis does offer a benefit in terms of recovery of renal function.

Another strategy that has been considered in the management of methotrexate nephrotoxicity is continuous renal replacement therapy (CRRT), which provides more continuous clearance than intermittent or high-flux hemodialysis over a longer period of time. Therefore, rebound in toxin levels is less likely to occur. Several cases in the literature have documented a reduction in serum methotrexate levels using CRRT. For instance, a 79-year-old male with diffuse large B-cell lymphoma who had received intrathecal methotrexate developed a rise in serum creatinine from a baseline of 1.08 to 3.59 mg/dl, in spite of dosage reductions, intravenous fluid hydration, urinary alkalinization, leucovorin rescue, and a reduction in serum methotrexate levels from 59.05 umol/L to 0.81 umol/L. In addition, he developed pancytopenia, and his serum methotrexate levels again rose from 0.51 to 0.63 umol/L. The patient also developed evidence of volume overload. Continuous veno-venous hemofiltration (CVVH), which generally involves more fluid and solute removal through convection rather diffusion, was therefore initiated using a high-flux polyethersulfone filter with a membrane surface area of 1.5 m^2^ and a standard blood flow rate of 300 ml/min. The serum prefilter and postfilter methotrexate concentrations were 0.74 and 0.58 umol/L, respectively. As his renal failure and volume overload persisted, the patient was transitioned to continuous veno-venous hemodiafiltration (CVVHD). Ultimately, the patient developed an acute-on-chronic subdural hematoma resulting from the CNS lymphoma, and eventually, comfort care measures were initiated and the patient expired. CVVH had been initiated 10 days after methotrexate administration [4]. The study raises the question of whether the timing of CRRT initiation may have influenced the outcome. It is possible that initiation of CRRT 10 days following methotrexate administration may have led to delayed removal of the drug and therefore reduced the likelihood of recovery of renal function. The patient had developed AKI, which probably influenced mortality in this case. It is possible that development of the subdural hematoma may have occurred through a systemic inflammatory response, which significantly influences morbidity and mortality in patients with both AKI and chronic kidney disease.

Another study examined the role of CVVH in combination with charcoal hemoperfusion for methotrexate removal. A 64-year-old female with diffuse large B-cell lymphoma had initially received methotrexate 8 g/m^2^ due to rapid progression of her disease. She had received standard management to prevent methotrexate nephrotoxicity but required 11 days to serum methotrexate concentrations to reach nontoxic levels (less than 0.1 umol/L). Thirty days later, however, she received a second dose of methotrexate. Even though she received appropriate intravenous fluid hydration, urinary alkalinization, and a higher than standard dose of leucovorin, her methotrexate concentrations reached toxic levels of 437.5 umol/L. The patient developed stomatitis, thrombocytopenia, and her serum creatinine rose to 2 mg/dl from a baseline of 1.1 mg/dl. The patient received a four-hour course of charcoal hemoperfusion, which is often effective for removal of plasma protein-bound molecules such as methotrexate followed by CVVH. A standard blood flow rate of 300 ml/min was used. Following charcoal hemoperfusion, serum methotrexate levels dropped to 362 umol/L, then to 160 umol/L following eight hours of CVVH. After 96 hours of CVVH, serum methotrexate levels were 3.55 umol/L, but the rate of decline in serum methotrexate levels decreased. The effluent methotrexate levels were 0.7 umol/L, which was less than the urinary methotrexate levels of 47.8 umol/L. Therefore, the clinicians felt forced diuresis would be more effective than CVVH for methotrexate removal and thereby stopped CRRT. While the urine output did increase with diuresis, serum methotrexate levels again rose from 1.2 to 1.97 umol/L within 24 hours. After CVVH was restarted, serum methotrexate levels did again drop to 0.53 umol/L but prognosis at this point was poor due to the lymphoma and complications from methotrexate toxicity. Therefore, CVVH was stopped and comfort care measures were instituted [7]. While CRRT does have the advantage of avoiding rebound of serum methotrexate levels due to being continuous over a long period of time and also of less likelihood of hypotension, in comparison with intermittent hemodialysis, due to slower blood flow rates and more gentle fluid removal, it did not appear to provide a mortality benefit nor a benefit in terms of renal recovery in either case described above. However, CRRT is usually initiated in hemodynamically unstable patients, which already predisposes to a greater likelihood of morbidity and mortality, not to mention lower likelihood of renal function recovery. While hypotension is less likely with CRRT, it is not entirely avoidable. Therefore, in some cases, renal hypoperfusion can still occur, limiting the recovery of renal function.

High-dose methotrexate and its role in ocular lymphoma

MTX can be administered intravenously at high dose and/or intrathecally in addition to intravitreal administration [8]. Radiation therapy may also be used for central nervous system disease. Recent studies examined the effectiveness of a sustained-release methotrexate implant for primary intraocular lymphoma in rabbit eyes with the targeted therapeutic range of 0.1 to 1.0 micromolar; however, there were difficulties encountered with a sustained release of methotrexate over prolonged periods of two to three months [9]. The optimal treatment for isolated primary intraocular lymphoma is limited to retrospective case reports or mostly small series with heterogenous patient populations and treatment approaches. Primary isolated intraocular lymphoma has been treated in one prospective trial using a combination of external beam radiation and methotrexate (3.5 g/m^2^) intravenously over four hours followed by leucovorin rescue, vincristine and procarbazine given every two weeks for six cycles [10]. Patients underwent follow-up evaluations every three to six months and annual surveillance with brain magnetic resonance imaging (MRI). High dose methotrexate 3.5 g/m^2^ and/or high-dose cytarabine at 3g/m^2^ or higher can be combined with ocular radiation therapy leading to remission in several studies [11].

## Conclusions

To summarize the evaluation of systemic toxicities in DLBCL patients receiving high dose methotrexate is complex and requires a multidisciplinary approach. With respect to evaluation of renal toxicity, glucoparidase, high-flux hemodialysis, and CRRT may be considered in the management of methotrexate nephrotoxicity in certain cases. Glucoparidase will most likely lead to reduction in serum methotrexate concentrations and recovery of renal function if administered early. It is uncertain whether glucoparidase is more effective in terms of renal function recovery than renal replacement therapy as no head-to-head comparisons have been done. However, in cases where the patient develops uremia, electrolyte imbalances, or volume overload, glucoparidase alone will not be sufficient. High-flux hemodialysis or CRRT is advised in those cases. High-flux hemodialysis should be reserved for hemodynamically stable patients and CRRT for patients who are hemodynamically unstable. In the case of high-flux hemodialysis or CRRT, removal of methotrexate will likely occur. Also, high-flux hemodialysis or CRRT will prevent fluid overload, electrolyte and acid-base imbalances, and allow removal of uremic toxins. However, it is not guaranteed that high-flux hemodialysis or CRRT will enhance recovery of renal function, and therefore more long-term studies with a larger number of patients need to be done. Also, as demonstrated in one of the cases, rebound in serum methotrexate levels may still occur even after CRRT is stopped. Glucoparidase should ideally be administered before the patient develops evidence of volume overload, electrolyte or acid-base imbalances, or uremic symptoms. Selection of any of the above-mentioned management strategies must be tailored to the individual patient. With respect to systemic effects, we must monitor acute neurological toxicities in addition to monitoring a patient's intravascular volume status, as it plays a key role in drug clearance and symptom control as it pertains to the chemoreceptor trigger zone (CTZ) and emetic centers, all which involve dopaminergic and vagosympathetic receptors. By understanding acute MTX toxicity on a multitude of physiologic and pharamacologic levels we are better able to deliver higher quality healthcare with enhanced compliance and therapeutic outcomes.
